# Transfer RNA-derived small RNAs in tumor microenvironment

**DOI:** 10.1186/s12943-023-01742-w

**Published:** 2023-02-16

**Authors:** Mei Yang, Yongzhen Mo, Daixi Ren, Shun Liu, Zhaoyang Zeng, Wei Xiong

**Affiliations:** 1 NHC Key Laboratory of Carcinogenesis and Hunan Key Laboratory of Cancer Metabolism, Hunan Cancer Hospital and the Affiliated Cancer Hospital of Xiangya School of Medicine, Central South University, Changsha, China; 2 Key Laboratory of Carcinogenesis and Cancer Invasion of the Chinese Ministry of Education, Cancer Research Institute, Central South University, Changsha, China; 3 Department of Cardiovascular Medicine, the Second Xiangya Hospital of Central South University, Changsha, China

**Keywords:** Transfer RNAs, tRNA-derived small RNAs (tDRs), tRFs, tiRNAs, Tumor microenvironment (TME), Biomarkers, Therapeutic

## Abstract

Transfer RNAs (tRNAs) are a class of non-coding RNAs responsible for amino acid translocation during protein synthesis and are ubiquitously found in organisms. With certain modifications and under specific conditions, tRNAs can be sheared and fragmented into small non-coding RNAs, also known as tRNA-derived small RNAs (tDRs). With the development of high-throughput sequencing technologies and bioinformatic strategies, more and more tDRs have been identified and their functions in organisms have been characterized. tRNA and it derived tDRs, have been shown to be essential not only for transcription and translation, but also for regulating cell proliferation, apoptosis, metastasis, and immunity. Aberrant expression of tDRs is associated with a wide range of human diseases, especially with tumorigenesis and tumor progression. The tumor microenvironment (TME) is a complex ecosystem consisting of various cellular and cell-free components that are mutually compatible with the tumor. It has been shown that tDRs regulate the TME by regulating cancer stem cells, immunity, energy metabolism, epithelial mesenchymal transition, and extracellular matrix remodeling, playing a pro-tumor or tumor suppressor role. In this review, the biogenesis, classification, and function of tDRs, as well as their effects on the TME and the clinical application prospects will be summarized and discussed based on up to date available knowledge.

## Introduction

Transfer RNAs (tRNAs) are a class of highly structured and heavily modified non-coding RNA molecules transcribed from tRNA genes [1]. In eukaryotes, tRNA genes are transcribed by RNA polymerase III into precursor tRNAs, which are then further processed by specific enzymes [2]. The 5’-end leading sequence of the tRNA precursor is removed by the endonuclease RNase P [3], the 3’-end maturation including the 3’ tail sequence is removed by RNaseZ/ELAC2, and the CCA tail is added by a special nucleotidyl transferase (often called CCAse) [4, 5]. The mature tRNA contains 76 nucleotides (nts) and has a “clover” shaped secondary structure consisting of four arms and three loops. The four arms are the acceptor stem, D arm, TΨC arm, and anticodon arm; and the three loops are the D loop, TΨC loop, and anticodon loop. In addition, there is a variable loop structure at the connection of the anticodon arm and the TΨC arm. After further folding, a stable L-shaped tertiary structure is formed [1, 6]. tRNAs are mainly involved in protein synthesis by decoding the triplet codon on mRNAs and transporting specified amino acids.

tDRs are a novel class of small non-coding RNAs produced by tRNAs fragmentation [7], which were earlier thought to be the by-products of random degradation of tRNAs. With the development of deep sequencing technology and the refinement of bioinformatic analysis techniques, studies across species and thousands of samples have provided strong evidence for the importance of tDRs [8]. They perform a variety of valuable functions for the cell, such as involvement in gene silencing [9], ribosome genesis [10], translation efficiency [11], and epigenetic regulation [12, 13]. Meanwhile, tDRs have been demonstrated to be associated with a wide range of human diseases, including cancer [14], neurodegenerative diseases [15], metabolic diseases [16], and viral infections [17]. Especially, tDRs can regulate tumorigenesis and cancer development at multiple levels. In this paper, we will systematically review the biogenesis, classification, and biological functions of tDRs, and elaborate the impact of tDRs on TME and their possible clinical applications. Based on the rapid development of this field, a series of different tDRs nomenclatures have emerged, in order to standardize tDR names, this review adopts the tDRnamer system (http://trna.ucsc.edu/tDRnamer/) for uniform naming of the referenced tDRs [18].

### Classification and biogenesis of tDR

**Fig. 1:**
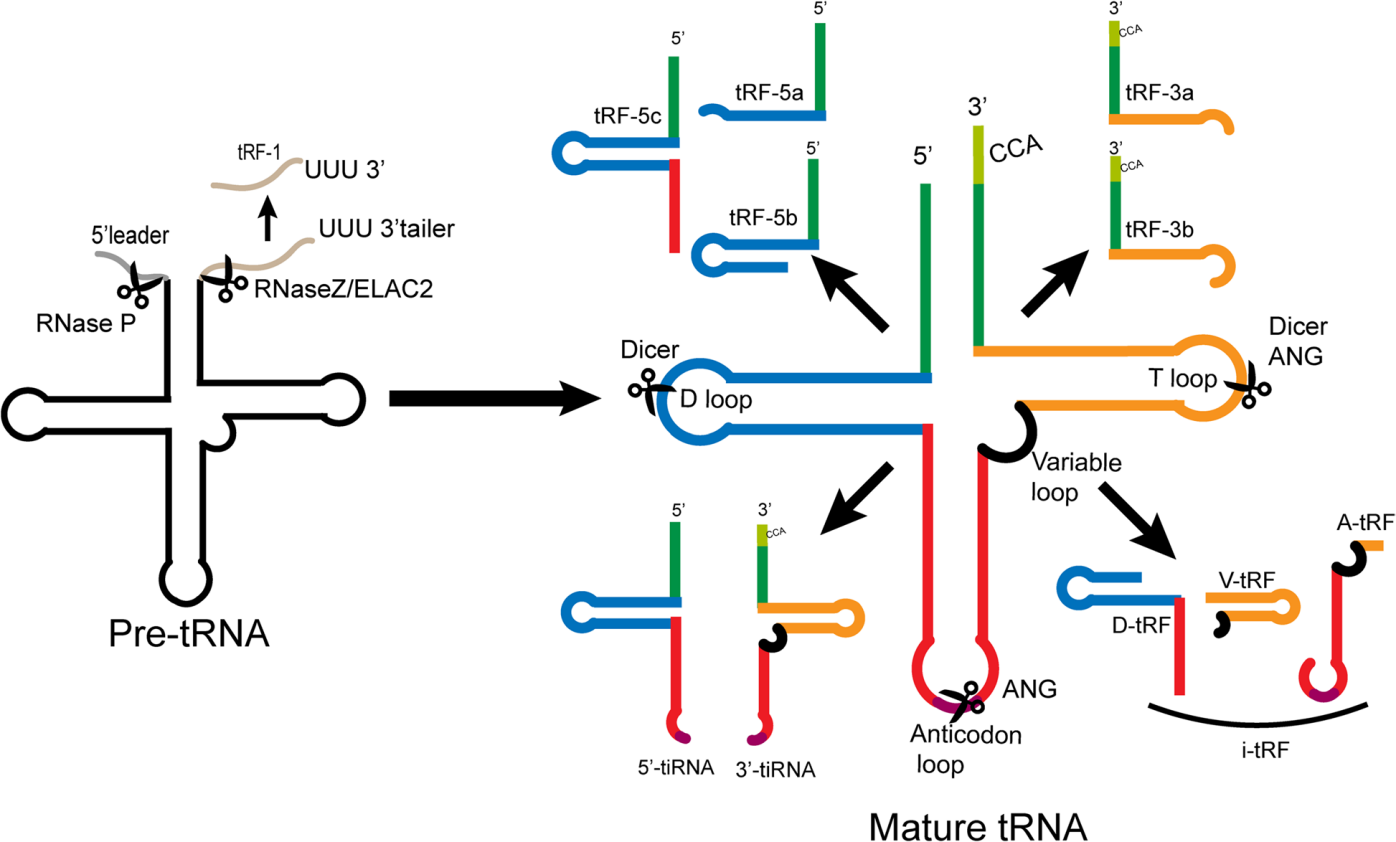
Biogenesis and classification of tDRs. During tRNA maturation, the 3’-trailer sequences are removed from pre-tRNA by the endonuclease Z (RNase Z/ELAC2), which results in the production of tRF-1. tiRNAs are generated by ANG cleavage of the anticodon loop of mature tRNAs under stressful conditions, including 5’-tiRNAs and 3’-tiRNAs. tRF-3 originates from the cleavage of the 3' end of the mature tRNA TѰC loop by Dicer or ANG. tRF-5 is cleaved in a Dicer-dependent manner in the D-loop or stem region of mature tRNA. Their subtypes are determined by the size and cleavage locations. i-tRFs are produced from the internal region of tRNAs, and can be classified into three types: D-tRF, A-tRF, and V-tRF, according to different cleavage regions

Based on the length and the site of origin of tDRs, they can be divided into two major categories: tRNA halves and tRNA-derived fragments (tRFs). Among them, tRNA halves, also known as tRNA-derived stress-induced RNAs (tiRNAs), which are 31–40 nts long RNA strands generated by angiogenin (ANG) responsible for cleaving the anticodon loop of mature tRNAs under stressful conditions (such as hypoxia, lack of amino acids, oxidative stress, ultraviolet radiation, heat shock, and viral infection) [19–22]. tiRNAs include 5’-tiRNAs and 3’-tiRNAs. 5’-tiRNAs have 30–35 nts from the 5’ end of the mature tRNAs to the anticodon loop, while 3’-tiRNAs have 40–50 nts from the anticodon loop to the 3’ end [22, 23]. Whereas, tRFs are derived from mature or pre tRNAs, 13–30 nts in length, and can be classified as tRF-1, tRF-3, tRF-5, and internal tRF (i-tRF) depending on their location of origin [24, 25]. tRF-1 is a tail sequence at the 3’ end of RNaseZ/ELAC2 cleaved pre-tRNA that is not contained in the mature tRNA sequence and has a poly U feature, hence named 3’ UtRFs, with a wide range of lengths [24]. tRF-3 (3’-tRF) originates from the cleavage of the 3' end of the mature tRNA TѰC loop by Dicer, ANG, or other RNase A superfamily members. They usually contain a CCA tail sequence and can be divided into tRF-3a (18 nts) and tRF-3b (22 nts) based on their lengths [23]. tRF-5 (5’-tRF) is cleaved in a Dicer-dependent manner in the D-loop or stem region of mature tRNA to produce fragments of different lengths: tRF-5a (14–16 nts), tRF-5b (22–24 nts), or tRF-5c (28–30 nts) [26]. In addition, the i-tRF generated from the internal region of the tRNA is named according to the starting position on the 5’ end in the tRNA. For example, D-tRF is formed by cleavage of the D-stem, A-tRF is formed by cleavage of the anticodon loop, V-tRF is generated by cleavage of the variable region, and the biological occurrence of i-tRF still needs further investigation [27] (Fig. 1). Interestingly, a distinction in the cellular localization of tDRs has been noticed, as we know that tRF-1 is primarily derived from cleavage of the 3’ tail of pre-tRNA in the nucleus, but is ultimately localized mainly in the cytoplasm. On the contrary, tRF-5 is formed by Dicer cleavage of mature tRNA in the cytoplasm, but is ultimately localized in the nucleus. The mechanism driving the differential localizations of tDRs remains unclear, which may be closely related to the execution of gene expression and protein translation regulatory functions [15, 28].

tDR biogenesis is tightly connected with tRNA modifications. It is well known that there are about 500 tRNA genes in the human genome that encode hundreds of tRNAs, but not all of them can be cleaved to produce tDRs. Eukaryotic RNA modifications have been known since the 1970s. To date, more than 170 chemical modifications have been identified, about 80% of which are decorated on tRNAs with an average of 11–13 modifications per tRNA in humans [29–31]. These modifications not only increase the stability of tRNA structure and alter its decoding function, but also affect tRNA fragmentation [32, 33]. Several anticodon tRNA modifications, namely Cm34 and Q34, are known to prevent ANG-mediated cleavage [34]. Additionally, the NOP2/Sun RNA methyltransferase family member 2 (NSUN2)-mediated m5C formation can also inhibit ANG-mediated tRNA cleavage [35]. In contrast, there are some modifications to promote tRNA cleavage, such as PUS7-mediated pseudouridine (ψ) activation of tRFs biogenesis [36]. These modifications control the production of tDRs under stress conditions.

### Biological functions of tDR

**Fig. 2:**
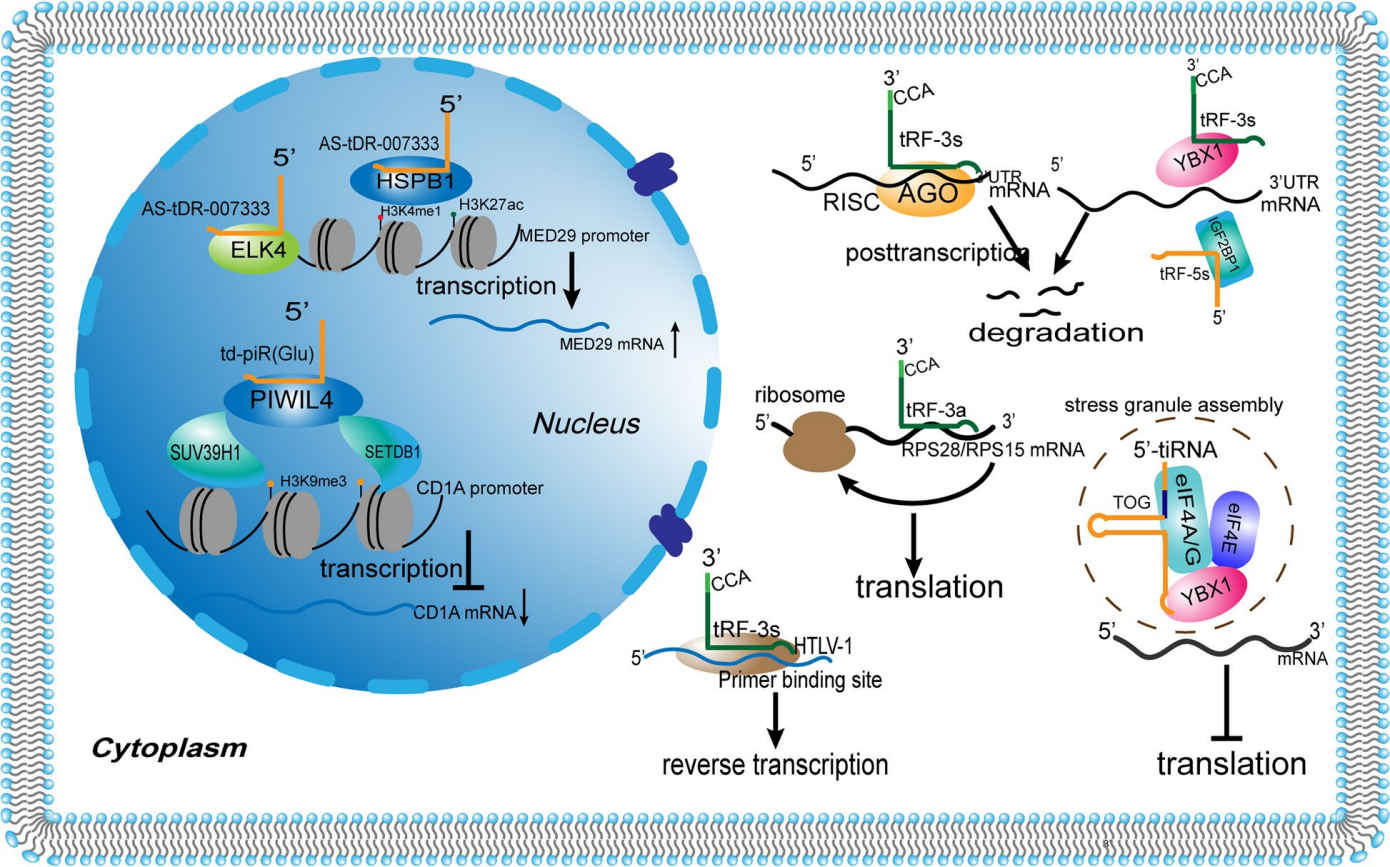
The working pattern of tDRs. Transfer RNA-derived small RNAs (tDRs) can regulate gene expression at multiple levels. In the nucleus, for example, AS‑tDR‑007333 interacts with HSPB1 protein that epigenetically augments MED29 transcription. td-piR (Glu), another tDR can recruit H3K9 methyltransferases (SETDB1 and SUV39H1) to the CD1A promoter region promoter region through binding PIWIL4 protein to form a complex, resulting in CD1A transcription inhibition. In the cytoplasm, tRF-3 s can interact with AGO proteins to induce the formation of silencing complexes (RISC) or act as protein decoy to isolate the binding of RBPs (YBX1 and IGF2BP1), thereby regulating mRNA stability at the post-transcriptional level. tiRNAs with the TOG motifs, assisted by YBX1, can inhibit the translation of target mRNA by substituting eIF4F complex and inducing SG assembly. tRFs can also bind two ribosomal protein (RPS28/RPS15) mRNAs to enhance their translation and regulate ribosome biogenesis. tRF-3 s can also promote reverse transcription by targeting the primer binding site (PBS) of the HTLV-1 in viral particles

tDRs are a class of multifunctional small non-coding RNAs. It is suggested that the localization of tDRs is closely related to their corresponding functions, and that the same type or same tDRs can perform different functions depending on its localization. While in the nucleus they can regulate gene expression at the pre-transcriptional and transcriptional levels, in the cytoplasm they can affect the stability of mRNA and inhibit or promote translation, they can also enter the viral particle and regulate reverse transcription [30, 37, 38]. In the following section, the functions of tDRs will be categorized according to their different localizations (Fig. 2).

### In the nucleus

#### Pre-transcriptional and transcriptional levels

tDRs regulate gene expression through epigenetic modifications. tDRs interact with PIWI proteins to exert piRNA-like functions. The tDR-1:29-Glu-CTC-1-M2 (td-piR (Glu)) from the 5’ end of mature tRNA^Glu^ is regulated by IL-4 and recruits histone methyltransferases in the CD1A promoter region of human monocytes by interacting with PIWIL4 to downregulate CD1A transcription through H3K9me3 methylation [37]. tRF-1 s, such as tDR-T1:T20-Thr-AGT-1–1 (ts-53) and tDR-T1:T25-Ser-GCT-4–3 (ts-101), interact with PIWIL2 proteins and act synergistically with DNA methyltransferases to affect DNA methylation and regulate pre-transcriptional gene expression [39, 40]. In addition to interacting with PIWI proteins, tDRs can also bind to other proteins to modify histone modifications and promote transcription. tDR-1:28-Gly-CCC-1-M4 (As-tDR-007333), a tDR derived from the 5’ end of tRNA^Gly(GCC)^ that is approximately 28nts long, binds to and interacts with HSBP1, mediates MED29 promoter region H3K4me1 and H3K27ac modifications to activate MED29 transcription, and also interacts with the transcription factor ELK4 to further modify the MED29 promoter to augment transcription of the oncogene MED29 (a subunit of regulatory Mediator (MED) complex) [41]. Boskovic et al. found that a 5’-tRF from tRNA^Gly(GCC)^ also regulates the production of other non-coding RNAs (U7 snRNA) and histone levels, affecting the conformation of heterochromatin and thus regulating the transcription of MERVL elements [42].

### In the cytoplasm

#### Posttranscriptional level

tDRs belongs to a group of small noncoding RNAs that possess sufficient sequence complementarity with endogenous mRNAs to play miRNA-like roles. tDRs can interact with AGO proteins to induce the formation of silencing complexes (RISC) and regulate mRNA stability by binding to the 3’ untranslated region of target genes, thereby regulating gene expression at the post-transcriptional level. Kuscu and his colleagues demonstrated that three tRF-3a species, namely tDR-59:76-Leu-AAG-1-M6 (tRF-3001a), tDR-60:76-Cys-GCA-2-M7 (tRF-3003a), and tDR-59:76-Leu-TAA-1 (tRF-3009a), repressed gene expression post transcriptionally in HEK293T cells in a Dicer-independent manner through an Argonaute-GW182 containing RISC via sequence matches with target mRNAs [43]. In another study, tDR-60:76-Cys-GCA-2-M7, produced by IL-1β-induced tRNA^Cys(GCA)^ cleavage, interacts with AGO2 in chondrocytes to induce RISC formation, post-transcriptionally regulates JAK3 expression and blocks pro-inflammatory signaling via the JAK/STAT pathway [44]. Recent studies in colorectal cancer revealed that a tRF from tRNA^Val^, which impairs FOXK1 mRNA stability and inhibits the Wnt/β-catenin pathway in an AGO-dependent manner, inhibited colorectal cancer cell proliferation and metastasis [45]. In addition, tDR-1:31-Glu-CTC-1-M2 (tRF5-GluCTC), generated by respiratory syncytial virus (RSV)-induced host tRNA^Glu(CTC)^ cleavage, represses the expression of the antiviral gene APOER2 mRNA through AGO1 and AGO4 protein-mediated recognition of the 3’-portion of the APOER2 3' -UTR, leading to viral replication [46, 47].

tDRs can also act as protein decoy to isolate RNA-binding proteins (RBPs) from target RNA, thereby affecting RNA stability. YBX1 is a multifunctional RBP that binds to a variety of oncogenic transcripts, stabilizing and enhancing transcript expression. While hypoxia-induced tRFs from tRNA^Glu^, tRNA^Asp^, tRNA^Gly^, and tRNA^Tyr^ can substitute YBX1 for binding to oncogenic transcripts in breast cancer, resulting in destabilization of these oncogenic transcripts and tumor suppression through post-transcriptional mechanisms [48]. In addition, IGF2BP1, another RBP, was found to bind to tDRs and regulate the stability and translation of a variety of mRNAs, including c-Myc. In retinoic acid (RA)-induced differentiated mouse embryonic stem cells, 5’-tiRNAs (from tRNA^Gln(CTG)^, tRNA^Glu(TTC)^, tRNA^Val(AAC)^, and tRNA^Gly(GCC)^) can disrupt IGF2BP1 binding to c-Myc mRNA and affect the stability of c-Myc mRNA through preferentially binding to IGF2BP1 [49].

#### Translation level

tDRs can affect translation by regulating translation initiation, elongation, and ribosome genesis, ultimately affecting protein production. When facing stressful environments, such as hypoxia, oxidative stress, or nutritional deficiency, cells protect themselves by inducing the cleavage of tRNAs to generate 5’-tiRNAs (not 3’-tiRNAs) that inhibit translation until the transient stress wanes. These tiRNAs with 5’-terminal oligoguanine (TOG) motifs possess the ability to displace the eIF4F complex, induce stress granule (SG) assembling, resulting in the failure of the translation initiation scan step, thereby inhibiting translation, and the tiRNA-associated translational silencer YBX1 contributes to the process of stress-induced translational repression [50–52]. By contrast, Sobala A et al. found that 5’-tRFs, which requires two guanosine residues (G18 and G19) at the 3’ end of the molecule, unlike the G-quadruplex (G4) structure at extreme 5’ end of tiRNAs [51], did not affect translation initiation but were associated with polyribosome extension, thereby inhibiting the extension phase of translation [53]. Similar phenomena were observed in Arabidopsis plants [54]. Both in human cells and Arabidopsis, these tDRs were shown to independent of AGO protein and mRNA sequences, thus suggesting that they work in a completely different pathway from the gene silencing [53, 54]. Nevertheless, tDRs can also promote protein synthesis by enhancing translation-induced ribosome biogenesis. A specific 3’-tRF from tRNA^Leu(CAG)^ can bind at least two ribosomal protein (RPS28 and RPS15) mRNAs to enhance their translation and regulate ribosome biogenesis [10].

#### Reverse transcription level

Different roles of tDRs in reverse transcription have also been identified. tDR-59:76-Pro-AGG-1-M8 (tRF-3019) from the 3’ end of tRNA^Pro^ was identified in the repertoire of small non-coding RNAs expressed by normal T cells compared to cells transformed with human T-cell leukemia virus type 1 (HTLV-1). tDR-59:76-Pro-AGG-1-M8 (tRF-3019) can be wrapped into the viral particles with perfect sequence complementarity to the primer binding site (PBS) of HTLV-1 and primes HTLV-1 reverse transcriptase [55]. However, Schorn and his colleagues uncovered the opposite role of tDRs in reverse transcription [56]. When endogenous retrovirus (ERV) is released from epigenetic silencing, 3’-tRFs can strongly inhibit the long terminal repeat (LTR)-retrotransposon or ERVs activity in mouse stem cells by targeting the highly conserved primer binding site (PBS). The 18-nt tRF-3 (tRF-3a) inhibits retrotransposition by competing for binding of the PBS with full-length tRNAs to block reverse transcription, while 22-nt tRF-3 (tRF-3b) mediates post-transcriptional silencing of MusD via RNA interference by targeting the PBS [56]. Recent studies show that TRMT6/61A-dependent m1A modifications can negatively affect the gene silencing function of 3’-tRFs, which further reveals the upstream regulatory mechanism of tDRs [57].

### Role of tDR in tumor microenvironment

Aberrant expression of tDRs has been found in many diseases, including viral infections [46], neurodegenerative diseases [15, 58], metabolic diseases [16], and cancer [14, 48, 59]. Its relationship with cancer has attracted great interest. Tumor microenvironment (TME), as a complex ecosystem on which tumors depend, is often referred to as a soil-to-seed relationship and consists mainly of cellular and cell-free components, including various stromal cells such as immune cells (cytotoxic T cells, regulatory T cells, myeloid-derived suppressor cells, tumor-associated macrophages), tumor-associated fibroblasts, endothelial cells, mast cells, and cell-free components mainly including blood vessels, lymphatic vessels, extracellular matrix, and soluble proteins [60–62]. tDRs serve as a bridge for information communication between cells and their surroundings, and plays a key role in tumorigenesis [60]. Hypoxia, oxidative stress, high lactate, and inflammatory cytokines in the TME can induce or inhibit tDRs, which in turn can act on tumor cells and the surrounding environment. There is considerable evidence to indicate that tDRs are involved in the development of cancer, through regulating tumor stem cells, energy metabolism, immune infiltration, angiogenesis, epithelial mesenchymal transition, and extracellular matrix remodeling in the microenvironment, thus exerting pro- or anti-tumor effects (Table 1).

**Table 1:** Regulatory tDRs in tumor microenvironment

tDR Name	tDR Type	Regulation Level	Mechanism	Function	Cancer Type	References
tRF/miR-1280	tRF-3	posttranscription	target JAG2 to suppress Notch/Gata signaling and up-regulate miR-200b	inhibit CRC stem cell phenotype and EMT	Colorectal cancer	[59]
5'tRNA fragments	tRF-5	translation	inhibit protein synthesis	increase tumor initiation population and promote tumor stem cell function	Skin cancer	[63]
TOG-containing 5’-tRFs	tRF-5	translation	inhibit protein synthesis	promote stem cell commitment	Hematological malignancies	[36]
ts-34 ts-49	tRF-1	/	/	associate with T cell activation status	Breast cancer	[64]
5'-tRF^Cys^	5'-tiRNA	posttranscription	promote oligomerization of Nucleolin and protect metabolic transcripts Mthfd1l and Pafah1b1 from exonucleolytic degradation	promote metastasis	Breast cancer	[65]
tRF^Lys−CTT−010^	tRF-5c	posttranscription	target G6PC regulates glucose metabolism	promote the proliferation and metastasis of TNBC	Breast cancer	[66]
5'-HisGUG Half	5'-tiRNA	posttranscription	selective binding to AGO2 after LA induction and increased their stability	promote the proliferation of B-cell lymphoma cells	B-cell lymphoma	[67]
tRF-20- M0NK5Y93	i-tRF	posttranscription	target Claudin-1	down-regulate EMT-related markers and suppress CRC cell invasion and metastasis	Colorectal cancer	[68]
Gly-tRF	5'-tRF	posttranscription	target NDFIP2 and activate AKT signaling pathway	enhance LCSC-like cell characteristics and promote EMT	Liver cancer	[69]
miR-720	tRF	posttranscription	target Rab35	induce EMT of cervical cancer cells and promote cell migration	Cervical cancer	[70]
tRF-21	i-tRF	/	LIF or IL-6 inhibits its binding to hnRNPL and prevent phosphorylation of hnRNPL by AKT2/1	repress tumor growth and metastasis	PDAC	[71]
tRiMetF31	5'-tiRNA	posttranscription	target PFKFB3	inhibit cell proliferation and angiogenesis	Neuroblastoma Breast cancer	[72] [73]

Abbreviations: *CRC* Colorectal cancer, *TNBC* Triple negative breast cancer, *LCSC* Liver cancer stem cells, *PDAC* Pancreatic ductal adenocarcinoma, *EMT* Epithelial-mesenchymal transition

#### tDR and cancer stem cells

Cancer stem cells (CSC) are an important component of the TME. tDR acts as an essential intermediate molecule that affects cancer stem cells or tumor initiating population in tumorigenesis. NSUN2 deficiency leads to accumulation of 5’-tRFs that inhibit translation and protein synthesis, increases tumor initiation population, and promotes stem cell function, leading to tumorigenesis [63], whereas methyltransferase NSUN2-mediated methylation (m5C) could protect tRNA from cleavage to maintain global translation [74]. Another PUS7-mediated pseudoureylation (ψ) modification activates the biogenesis of tDRs, the pseudouridylated TOG-containing 5’-tRFs control protein synthesis and stem cell fate through the post-transcriptional regulatory network in tumorigenesis [36]. tRF/miR-1280, acting as a key regulator of CSC growth and function in colorectal cancer cells, inhibits the stem cell phenotype of colorectal cancer by directly interacting with the JAG2 3’ UTR to suppress the Notch/Gata signaling, resulting in inhibiting the growth and metastasis of colorectal cancer [59].

#### tDR and tumor immunity

An interesting early study found that the largest human tRNA gene cluster is in the major histocompatibility complex (MHC), a genomic region critical in adaptive and innate immunity, which suggests a role for tRNA in the immune system [75]. In deep sequencing, small RNAs from the 5’ and 3’ termini of mature tRNAs were found to be abundant in the cytoplasm of immune cells, with predominantly small RNAs from the 5’ termini being selectively enriched in the vesicles of these immune cells and secreted by immune cells as immune signaling molecules to the extracellular compartment and play a role in the immune response [76–78]. This suggests an important role for parental tRNAs and their derived fragments in immunity. A previous study showed the effect of different fragments from tRNA^Ala(UGC)^ on the immune response in hepatitis B virus (HBV) infection, concluding that the 3’CCACCA sequence of tRNA^Ala(UGC)^ is the important motif to induce Th1 and cytotoxic T cell (CTL) responses and this motif can be effectively recognized by TLR [79].Subsequently, Chiou et al. showed that activated T cells use the extracellular vesicles (EVs) biogenesis pathway to selectively secrete tRFs that inhibit T cell activation, thereby promoting T cell activation and cytokine production [80]. The role of tDRs in the immune response was further revealed, but their study of the effect of tDRs on the immune status of tumors in the TME is still at a preliminary stage. Studies from Shan et al. using a large cohort study available in TCGA, revealed that tDR-T1:T18-Val-TAC-3–1 (ts-34) and tDR-T1:T34-Thr-CGT-2–1-A15G (ts-49) were significantly associated with T cell activation status. The levels of tDR-T1:T18-Val-TAC-3–1 and tDR-T1:T34-Thr-CGT-2–1-A15G in the T cell depleted group were tightly associated with overall survival breast cancer patients [64]. These studies indicate a promising role of tDR in the regulation of tumor immunity in TME.

#### tDR and energy metabolism

Metabolic reprogramming is considered as a hallmark of cancer [81]. tDRs may play important roles on the crossroad between metabolism and TME. A recent study reveals that tDR-1:34-Cys-CGA-2-M5 (5’-tRF^Cys^) can promote oligomerization of Nucleolin, an RNA-binding protein, into a transcript stabilizing ribonucleoprotein complex, thereby driving specific metabolic pathways underlying breast cancer metastatic progression [65]. Genome-wide transcriptome analysis of small non-coding RNA showed that tDR-1:31-Lys-CTT-1-M3 (tRF^Lys−CTT−010^) was significantly increased in human triple negative breast cancer (TNBC), and knockdown of tDR-1:31-Lys-CTT-1-M3 inhibited TNBC cell proliferation, migration, and invasion [66]. Further study showed that tDR-1:31-Lys-CTT-1-M3 was closely associated with the starch and sucrose metabolic pathway and positively regulated the expression of the catalytic subunit of glucose-6-phosphatase, suggesting that tDR-1:31-Lys-CTT-1-M3 promoted tumor growth and metastasis by reprogramming glucose metabolism through G6PC, with upregulation of lactate (LA) levels and downregulation of glycogen levels [66]. Meanwhile, in the B-cell lymphoma microenvironment, high levels of lactate (LA) enhance the expression of RNA polymerase III and ANG-mediated production of tDR-1:33-His-GUG-1-M9 (5’-HisGUG Half) through selectively binding to AGO2. High expression of tDR-1:33-His-GUG-1-M9 enhanced the stability of the respective and promoted the proliferation of B-cell lymphoma cells. When tDR-1:33-His-GUG-1-M9 was inhibited in vitro and in vivo, the growth of LA-induced B-cell lymphoma was reduced [67]. These suggest that tumor cell metabolism can lead to the accumulation of metabolites in the microenvironment that induce the production of tDRs, which in turn regulate tumor progression via metabolic pathways, elucidating the interplay between tDRs, energy metabolism, and tumor cells forms a feedback loop in the TME.

#### tDR and epithelial mesenchymal transition and extracellular matrix remodeling

Epithelial-mesenchymal transition (EMT) has been closely associated with cancer metastasis as the change in phenotype from epithelial to mesenchymal allows cancer cells to become invasive [82]. Functional studies have confirmed that tDRs can play multifaceted roles in tumor metastasis by regulating the EMT progress and extracellular matrix remodeling. Luan et al. identified a human-specific tRF, tDR-35:54-Arg-CCG-1 (tRF-20-M0NK5Y93), acting as an tumor suppressor, could directly target downstream Claudin-1 to downregulate EMT-related markers, resulting in inhibition of colorectal cancer (CRC) cell invasion and metastasis [68]. In addition, tRF/miR-1280 was reported to inhibits the Notch/Gata signaling pathway by directly interacting with the JAG2 3’ UTR, and simultaneously upregulates miR-200b expression, thereby inhibit the EMT and extracellular matrix degradation of colorectal cancer [59]. Apart from the role of tDRs in CRC metastasis, other cancer types also exhibit tDRs dysregulation. for example, tDR-1:30-Gly-GCC-2-M10 (Gly-tRF), which was highly expressed in hepatocellular carcinoma (HCC) cell lines and tumor tissues, could activate the AKT signaling pathway to enhance liver cancer stem cells (LCSC)-like cell properties and promote the EMT progress through targeting NDFIP2 mRNA 3’ UTR in a miRNA-like manner [69]. In cervical cancer, overexpression of miR-720 can lead to decreased E-cadherin and increased waveform protein levels by targeting Rab35 to promote EMT during cancer metastasis [70]. Interestingly, it is reported that miR-720 is not a classic miRNA, but is probably a fragment of tRNA [83]. The important role of tDRs in EMT suggests that tDRs could be attractive targets for novel therapeutic approaches.

#### tDR and inflammatory cytokines

Inflammatory cytokines act as an important class of tumor-promoting signaling molecules in TME [81]. Long-term stimulation of inflammatory cytokines may induce cellular malignancy and accelerate tumor progression. Interestingly, the biogenesis of tDRs can be regulated by certain cytokines, thus affecting tumor development and progression. Pan et al. identified an inflammatory cytokine-regulated tRF (tDR-19:39-Gly-GCC-2-M10 or tRF-21) from tRNA^Gly(GCC)^ by the splicing factor SRSF5 could be a tumor suppressor in pancreatic ductal adenocarcinoma (PDAC). tDR-19:39-Gly-GCC-2-M10 prevents phosphorylation of hnRNP L by AKT2/1 through binding to Ser52 of the Gly-rich structural domain of the oncogenic RNA-binding protein hnRNP L, which attenuates the formation of hnRNP L-DDX17. Upon stimulation with LIF or IL-6 in PDAC TME, the transcriptional suppressor KLF4 can repress tDR-19:39-Gly-GCC-2-M10 formation from tRNA^Gly(GCC)^ in a dose-dependent manner by binding to the promoter of the splicing factor SRSF5 in PDAC cells [71].

#### tDR and angiogenesis

Angiogenesis plays a key role in supporting tumor growth and metastasis. tDRs, as an emerging class of non-coding small RNAs, also plays an important role in tumor angiogenesis. For example, Li et al. found that fragments of tRNA^Val^ and tRNA^Gly^ origin could inhibit angiogenesis by regulating endothelial cell function in a rat brain ischemia model, a mouse hindlimb ischemia model, and a cellular hypoxia model, respectively [84]. On top of this, Wang and his colleagues demonstrated that a 31-nts tRNA^iMet^ fragment (tDR-1:31-iMet-CAT-1-M9 or tRiMetF31) from miR-34a-guided cleavage inhibited cell proliferation and angiogenesis by directly targeting PFKFB3 in neuroblastoma and breast cancer, which provides a new perspective on the inhibitory role of the miR-34a tumor suppressor network in tumor progression [72, 73]. Notably, the effects of tDRs on angiogenesis are still poorly understood and more studies are needed to clarify their role in angiogenesis.

### Perspectives of clinical application

Although pivotal roles of tDRs in TME has been discovered, due to its late start, only a tip of the iceberg is currently known. With the development of next generation sequencing technologies and bioinformatic strategies, researchers have found that tDRs are abundantly and stably distributed in body fluids and can be selectively encapsulated into exosomes, which allows them to be detected in a non-invasive and convenient manner [85]. In addition, tDRs are differentially expressed in a variety of tissues and cancers. Because of these properties, a growing number of studies have confirmed that tDRs can be used as potential biomarkers for diagnosis and prognosis of cancers, such as breast cancer [86], lung cancer [41], gastric cancer [87], colorectal cancer [88], pancreatic cancer [89], hepatocellular cancers [90], and ovarian cancer [91]. As we know, unlimited proliferation of cancer cells, evasion of apoptosis, invasion and metastasis, immune escape, sustained angiogenesis, tumor pro-inflammation, deregulating cellular energetics, all these hallmarks are dependent to varying degrees on TME. With increasing understanding of the vital role of TME in tumor development and treatment resistance, targeting TME has been found to offer significant therapeutic advantages other than direct targeting of cancer cells [92]. The use of immune checkpoint blockers (ICBs) and cellular therapies such as CAR-T cells in tumor immunotherapy has significantly improved clinical outcomes. The current clinical scenario for cancer patients faces two main obstacles: immune escape and acquired therapy resistance, both are associated with the immunosuppressive microenvironment, forcing us to look for new alternative strategies. Due to the key roles of tDRs in the cancer and TME, it provides a promising avenue to optimize and monitor treatments that could have real-time impacts on patient outcomes after miRNA [93] and circRNA [94] in the future. Nevertheless, research on tDRs is still at the early stage, there is still a long way to go before their clinical applications.

## Conclusions

In this review, we summarized evidence which showed that tDRs could regulate the TME in several dimensions. Studies aiming to unravel the mechanisms underlying these functions have shown that tDRs can be used not only as biomarkers for clinical diagnosis and prognosis, but also as potential targets for therapy. Targeting cancer cells directly or the TME indirectly are two major principles of anti-tumor strategies. Due to the heterogeneity of cancer cells and the instability of their genome, targeting the TME becomes a sensible choice. However, research on tDRs is just in its infancy, further explorations and discoveries are needed for a deeper understanding of the regulatory network of tDRs in TME. tDR-based monotherapy or combination therapy targeting the TME might be beneficial for cancer patients in the future.

## Authors’contributions

Mei Yang collected the related papers and drafted the manuscript. Yongzhen Mo, Daixi Ren, Shun Liu, Zhaoyang Zeng, and Wei Xiong participated in the design of the review and draft the manuscript. All authors read and approved the final manuscript.

## Data Availability

All data obtained and/or analyzed in this study were available from the corresponding author upon reasonable request.

## Abbreviations

tRNAs: Transfer RNAs
tDRs: Transfer RNA-derived small RNAs
tRFs: Transfer RNA-derived fragments
TME: Tumor microenvironment
RNase P: Ribonuclease P
RNaseZ/ELAC2: Ribonuclease Z/elaC ribonuclease Z 2
NSUN2: NOP2/Sun RNA methyltransferase family member 2
PUS7: Pseudouridine synthase 7
ANG: Angiogenin
PIWIL4: Piwi like RNA-mediated gene silencing 4
HSBP1: Heat shock factor binding protein 1
MED29: Mediator complex subunit 29
HTLV-1: Human T-cell leukemia virus type 1
PBS: Primer binding site
ERV: Endogenous retrovirus
RISC: RNA induced silencing complex
FOXK1: Forkhead box K1
APOER2: Apolipoprotein E receptor 2
YBX1: Y-box binding protein 1
IGF2BP1: Insulin like growth factor 2 mRNA binding protein 1
TOG: Terminal oligoguanine
G4: G-quadruplex
MHC: Major histocompatibility complex
TLR: Toll-like receptors
G6PC: Glucose-6-phosphatase, catalytic
EMT: Epithelial-mesenchymal transition
NDFIP2: Nedd4 family interacting protein 2
SRSF5: Serine and arginine rich splicing factor 5
PFKFB3: 6-Phosphofructo-2-kinase/fructose-2,6-biphosphatase 3
ICB: Immune checkpoint blocker
CAR-T cell: Chimeric antigen receptor T-Cell
